# Comparison between Clinical Utility of CXCL-8 and Clinical Practice Tumor Markers for Colorectal Cancer Diagnosis

**DOI:** 10.1155/2022/1213968

**Published:** 2022-12-16

**Authors:** Zhengyuan Huang, Zhaozhong Li, Xianqiang Chen, Xianjin Zhu, Junrong Zhang, Yanfang Song, Yingping Cao, Pingxia Lu

**Affiliations:** ^1^Department of Emergency Surgery, Fujian Medical University Union Hospital, 29 Xinquan Road, Fuzhou 350001, China; ^2^Department of Laboratory Medicine, Fujian Medical University Union Hospital, 29 Xinquan Road, Fuzhou 350001, China; ^3^Clinical Laboratory, Department of Laboratory Medicine, The Affiliated People's Hospital of Fujian University of Traditional Chinese Medicine, 602 Bayiqi Road, Fuzhou 350001, China

## Abstract

Owing to the high incidence and mortality rates of colorectal cancer (CRC), novel biomarkers for CRC diagnosis are critically needed. Therefore, this study is aimed at exploring the clinical utility of serum C-X-C motif chemokine 8 (CXCL-8) for CRC diagnosis and progression compared to the routinely used biomarkers, carcinoembryonic antigen (CEA), and carbohydrate antigen-19-9 (CA19-9). This study included 227 patients with CRC, 110 patients with colorectal adenoma (CA), and 123 healthy participants, who were recruited from the Fujian Medical University Union Hospital from July 1, 2019 to October 31, 2020. Serum concentrations of CXCL-8, CEA, and CA19-9 were detected using enzyme-linked immunosorbent assay and chemiluminescent microparticle immunoassay. Clinicopathological features of patients with CRC were collected and analyzed. The diagnostic efficacy of CXCL-8, CEA, and CA19-9 for CRC was evaluated using receiver operating characteristic (ROC) curves. We found that the serum concentrations of CXCL-8, CEA, and CA19-9 were significantly higher in patients with CRC than those in patients with CA and healthy controls. The diagnostic sensitivity of CXCL-8 alone was higher than those of CEA and CA19-9 both and when combined; thus, CXCL-8 may be better at discriminating patients with CRC from healthy controls and patients with CA. Moreover, combining CXCL-8 with CEA or CA19-9 improved their respective diagnostic performances in distinguishing patients with CRC from CA patients and healthy participants. Notably, we also found that serum concentrations of CXCL-8 were positively correlated with metastases and tumor size. Therefore, our study suggests that serum CXCL-8 may serve as an improved biomarker for CRC diagnosis compared to the traditional tumor markers CEA and CA19-9. Moreover, our findings indicate the potential efficacy of serum CXCL-8 levels as a CRC prognostic biomarker.

## 1. Introduction

Colorectal cancer (CRC) is one of the most common malignancies of the colon or rectum and has caused mass mortality in recent years [1, 2]. Approximately 881,000 people died from CRC in 2018, corresponding to more than 2400 cancer deaths on average per day worldwide [3]. Owing to economic growth, increasing urbanization, and lifestyle westernization, a substantial incidence and mortality rate has been observed in China, which imposes a heavy social and economic burden on individuals, families, and countries [4]. Colorectal adenomas (CA) are benign tumors of the colon or rectum and precursors of most CRCs, which take 5–10 years to develop into adenocarcinomas [5]. Moreover, it is difficult for clinicians to distinguish patients with CRC from patients with CA. Therefore, the early detection and diagnosis of CRC can reduce patient mortality and financial burden. Currently, several clinically useful methods, such as colonoscopy, rectoscopy, fecal occult blood test, and computed tomography, are used in the diagnosis of CRC; however, they have several limitations, such as invasiveness, high cost, low sensitivity, and low specificity [6, 7]. Alternatively, serum biomarker measurements can be used, as this method is less invasive and cheaper than others. However, the clinical practice tumor markers currently used have a poor diagnostic performance, especially for the early diagnosis of CRC [8]. Thus, it is essential to develop a minimally invasive, affordable, and, most importantly, more sensitive and specific approach for the early detection of CRC.

Many pathogenic factors are involved in CRC, and chronic inflammation is of utmost importance [9]. There is an increasing recognition that the inflammatory mediators and cellular effectors are important components of tumor microenvironment (TME). Regardless of its origin, inflammation in TME has a number of tumor-promoting effects [10]. Moreover, this cancer-related inflammation appears to be critical for better understanding the pathophysiology of cancer and the possibilities of its therapy and management [11]. Chemokines are a family of low-molecular cytokines which facilitate communication between tumor and nontumor cells within the TME. Many studies have shown that chemokines may facilitate neoplasm progression, which has made them the focus of investigations. Based on the positions of key cysteine residues, chemokines are grouped into four classes (CC, CX3C, CXC, and XC) [12]. C-X-C motif chemokine 8 (CXCL-8; also known as IL-8) is a small soluble peptide in the C-X-C chemokine family [13]. Early studies reported that CXCL-8 has many functions that promote cell proliferation, differentiation, migration, and survival [14]. Several studies have reported that serum CXCL-8 may represent a biomarker for esophageal [15, 16] and pancreatic cancers [17]. This suggests that serum CXCL-8 may be a candidate biomarker for certain tumors. Several studies have revealed the upregulation of CXCL-8 in CRC cells and CRC tissue and a correlation of CXCL-8 expression with a worse prognosis for patients with CRC [18, 19]. However, to the best of our knowledge, blood serum CXCL-8 has rarely been reported as a clinical biomarker for CRC diagnosis.

Therefore, our study aimed to detect the serum level of CXCL-8 in patients with CRC and compare this chemokine to current tumor markers to establish whether it may be considered an improved tumor marker for CRC diagnosis. Furthermore, we assessed whether CXCL-8 may be a potential candidate prognostic biomarker by evaluating the association between CXCL-8 and clinicopathological parameters in patients with CRC. Our findings suggest that CXCL-8 may be a potential biomarker for CRC diagnosis and progression.

## 2. Materials and Methods

### 2.1. Study Participants

Serum samples were collected from 227 patients (143 men and 84 women, median age (interquartile range): 61 (53–71) years) with newly diagnosed CRC. The controls included 110 patients with colorectal adenoma (CA; 67 men and 45 women, 56 (51–64) years), and 123 healthy participants (51 men and 72 women, 54 (41–65) years). All subjects were recruited from the Fujian Medical University Union Hospital (Fuzhou, China) between July 1, 2019 and October 31, 2020. The clinicopathological data of patients with CRC were recorded, including sex, age, tumor location, tumor size, tumor differentiation, TNM staging according to the American Joint Committee on Cancer classification guidelines [20], and nerve and vascular invasion. The main exclusion criteria were presence of infection, a history of cancer hyperpyrexia, pregnancy, haematological disease, intestinal obstruction or intestinal perforation at initial diagnosis, and incomplete information. This study was approved by the Institutional Medical Research Ethics Committee (2021KJCX013) of the Fujian Medical University Union Hospital (China). Informed consent for clinicopathological information and sample collection was provided by all participants.

### 2.2. Analysis of Serum CXCL-8, CEA, and CA19-9 Concentrations

Venous blood (5 mL) was obtained from all the participants. Venous blood samples from patients with CRC and CA were collected prior to any medical intervention. Serum was obtained by centrifugation at 3,000 rpm for 10 min and then stored at -80°C for subsequent measurements. The serum CXCL-8 concentration was measured using an enzyme-linked immunosorbent assay (R&D Systems Inc., Minneapolis, MN, USA) according to the manufacturer's instructions. A standard curve was constructed for each plate to calculate the absolute concentration. The serum concentrations of CA19-9 and CEA were measured using chemiluminescent microparticle immunoassay using a Cobas 6000 analyzer (Roche Diagnostics, Mannheim, Germany) according to the manufacturer's instructions. The cut-off value for normal CA19-9 was less than 37 U/mL and that for normal CEA was less than 5 ng/mL.

### 2.3. Statistical Analyses

Statistical analyses were performed using GraphPad Prism for window (version 5.0; GraphPad Software Inc., San Diego, CA, USA) or SPSS software for window (version 21.0; IBM, Armonk, NY, USA). The concentrations of the three markers did not conform to a normal distribution according to the normality test; therefore, nonparametric statistical analyses were applied [21]. The Mann–Whitney U test was performed to compare two groups, whereas the Kruskal-Wallis test was used for three or more group comparisons [21]. The diagnostic characteristics of CXCL-8, CEA, and CA19-9 were assessed using receiver operating characteristic (ROC) curves. The Youden index was used to determine the optimal cut-off value to differentiate between healthy controls, patients with CA, and patients with CRC. Combination analysis was performed using binary logistic regression. The relationship between the variables and the occurrence of CRC was evaluated using logistic regression; the odds ratio (OR) was adjusted for covariates [22]. The correlation between the serum concentrations of CXCL-8, CEA, and CA19-9 and the clinicopathological characteristics were determined using Spearman's rank method. The estimated coefficients were calculated by maximum likelihood method. The entry method was used for variable selection. The calibration was assessed via the Hosmer-Lemeshow goodness-of-fit test [22]. Differences were considered statistically significant at *p* < 0.05.

## 3. Results

### 3.1. Serum Concentrations of CXCL-8, CEA, and CA19-9 in Patients with CRC

**Figure 1:**
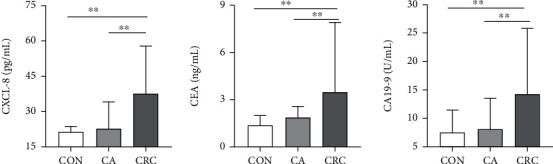
Serum concentrations of CXCL-8, CEA, and CA19-9 in patients with CRC, patients with CA, and healthy controls (CON). Data are presented as the median with the interquartile range. ^∗∗^*p* < 0.01.

Figure 1 shows the serum concentrations of CXCL-8, CEA, and CA19-9 in the clinical samples. The serum levels of all the markers were significantly higher (*p* < 0.001) in patients with CRC than in patients with CA and healthy participants.

### 3.2. Evaluation of Serum CXCL-8 as a Potential Biochemical Marker for CRC Diagnosis

We evaluated the clinical utility of CXCL-8 as a biochemical marker for CRC diagnosis compared to CEA and CA19-9, which are the most common serum biochemical markers in CRC diagnostics.

**Figure 2:**
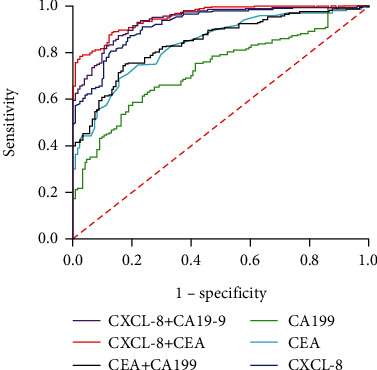
ROC curves of CXCL-8, CEA, and CA19-9 alone and combined for discriminating patients with colorectal cancer from healthy participants.

First, we evaluated the usefulness of CXCL-8 as a biomarker for the differential diagnosis between patients with CRC and healthy participants. The areas under the ROC curve (AUCs) for CXCL-8, CEA, and CA19-9 as parameters in CRC diagnostics were 0.920, 0.837, and 0.730, respectively (Figure 2 and Table 1). At a cut-off value of 24.92 for CXCL-8, we observed that the diagnostic sensitivity of CXCL-8 was higher (86.34%) than that of CEA (34.80%) and CA19-9 (15.86%) (Table 1). Our results also showed that CXCL-8 alone had better sensitivity and accuracy for discriminating patients with CRC from healthy controls than that of CEA and CA19-9 together (Table 1). Notably, the diagnostic utility of CEA and CA19-9 was improved when combined with CXCL-8 (Table 1). As shown in Figure 2 and Table 1, the AUCs for CXCL-8 + CEA and CXCL-8 + CA19-9 were 0.954 and 0.939, respectively, which were significantly higher than that for CEA + CA19-9 (AUC: 0.844). Moreover, the sensitivity and specificity of CXCL-8 + CEA and CXCL-8 + CA19-9 were considerably higher than those of CEA + CA19-9.

**Figure 3:**
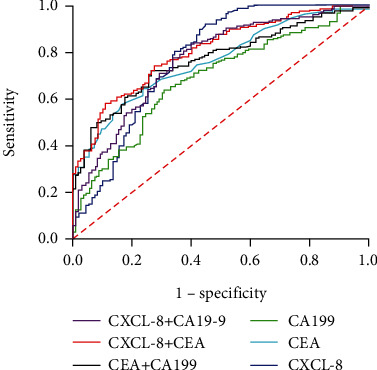
ROC curves of CXCL-8, CEA, and CA19-9 alone and combined for discriminating patients with colorectal cancer and patients with colorectal adenoma.

Next, we used ROC analysis to evaluate the efficacy of CXCL-8 in the differential diagnosis between patients with CRC and those with CA. As shown in Figure 3 and Table 2, the AUCs for CXCL-8, CEA, and CA19-9 were 0.774, 0.760, and 0.686, respectively, suggesting that CXCL-8 has no better diagnostic performance in distinguishing between CRC and CA. However, the diagnostic utility of CEA and CA19-9 was improved when combined with CXCL-8 (Figure 3 and Table 2). Notably, our results also showed that CXCL-8 alone had better sensitivity and accuracy for discriminating CRC from CA than that of CEA and CA19-9 alone or together (Table 2).

Taken together, these results indicate that serum concentrations of CXCL-8 may represent an improved novel biochemical marker for CRC diagnostics compared to clinical practice tumor markers.

### 3.3. Performance of CXCL-8, CEA, and CA19-9 for Predicting CRC Occurrence Risk via Cut-off Values

The correlation between several risk factors and CRC risk was initially evaluated using univariate analysis to identify the risk factors that qualified for the multivariate model (data not shown). All three markers were found to be associated with an increased risk of CRC occurrence and were entered into the multivariate analysis. Finally, only the serum concentrations of CXCL-8 and CEA (*p* = 0.000, OR = 7.76*p* = 0.001, and OR = 13.83, respectively) were significant risk factors for CRC occurrence (Table 3).

### 3.4. Association between Serum Concentrations of CXCL-8, CEA, and CA19-9 and Clinicopathological Characteristics in Patients with CRC

After determining the performance of serum CXCL-8 concentrations in assessing CRC, we further analyzed the association between the markers and the clinicopathological characteristics of the patients. The assessment of the relationship between the marker serum concentrations and the clinicopathological parameters revealed that serum concentrations of CXCL-8 increased with TNM stage, T stage, N stage, and nerve invasion, although these differences were not statistically significant (Table 4). However, these differences were statistically significant for the serum CEA levels (Table 4). For the M stage, serum levels of all the markers were found to be significantly higher in the patients with distant metastases (M1) than in patients without distant metastases (M0, *p* < 0.05) (Table 4). Additionally, patients with CRC with a tumor size ≥5 cm showed significantly higher CXCL-8 and CEA concentrations than those with a tumor size <5 cm (*p* < 0.05) (Table 4). However, no significant associations were observed between any of the marker levels and location, histological grade, or vascular invasion.

Correlations between the marker serum concentrations and clinicopathological characteristics of malignancy were assessed using the Spearman's rank correlation test (Table 5). Serum CXCL-8 levels were significantly correlated with the M stage (*p* = 0.039) and tumor size (*p* = 0.034) in patients with CRC (Table 5). Taken together, these findings indicate that serum CXCL-8 concentration is associated with an advanced clinicopathologic status in patients with CRC (Table 6). Table 6 shows all the acronym and full name in the article.

## 4. Discussion

In this study, we found that serum concentrations of CXCL-8 were significantly higher in patients with CRC than in patients with CA and healthy controls. Serum CXCL-8 alone had a better diagnostic sensitivity and accuracy in distinguishing CRC patients from CA patients and healthy participants than CEA and CA19-9 alone or in combination. Moreover, the addition of CXCL-8 improved the diagnostic sensitivity of CEA and CA19-9 in distinguishing patients with CRC from CA patients and healthy controls. Furthermore, serum CXCL-8 levels were significantly associated with M stage and tumor size.

CXCL-8 is a small soluble C-X-C chemokine that functions in chronic inflammation and cancer development [13, 14]. Many investigations have indicated that CXCL-8 plays an important role in tumor angiogenesis and invasion and is linked with distant metastases in CRC [23–25]. However, these studies have mainly assessed the expression levels of chemokines in CRC cell lines and tissues. To the best of our knowledge, serum CXCL-8 has rarely been reported as a biochemical marker for the diagnosis and prognosis of CRC.

CRC remains a severe global problem as it is a life-threatening malignancy with high morbidity and mortality. Therefore, recent studies have focused on improving early diagnostic methods for CRC. Endoscopy is currently the gold standard for the diagnosis of CRC; however, it had the lowest patient compliance rate owing to bowel preparation requirements and discomfort during the test. Furthermore, patients with serious cardiopulmonary insufficiency, intestinal perforation, or enterostenosis cannot undergo invasive tests [26, 27]. In general, serum biochemical marker measurements impose minimal inconvenience compared to endoscopy and provide lower financial expenses for patients. However, the diagnostic performance of the current clinical practice tumor markers, CEA and CA19-9, has been demonstrated to be poor, especially for stratifying the early stages of CRC [28, 29], which was further confirmed in our study. Our data showed that CXCL-8 had better diagnostic sensitivity and accuracy than CEA and CA19-9 alone or together for CRC detection. Notably, our findings also indicated that the combined use of CXCL-8 with CEA or CA19-9 improved their individual performances for CRC diagnosis. Moreover, the AUC for CXCL-8 (0.920) was significantly higher than that in a previous report (0.778) [30], in which there were 59 patients with CRC and 46 healthy participants. Moreover, CA is precursors of most CRC, and it is difficult to make a differential diagnosis between patients with CRC and patients with CA [28]. Notably, we found for the first time that CXCL-8 had a better diagnostic sensitivity and accuracy CEA and CA19-9 alone or together in differentiating CRC from CA. Additionally, our results indicate that the combined use of CXCL-8 with CEA or CA19-9 improved their diagnostic value. Notably, like CEA, serum CXCL-8 levels were a significant risk factor for CRC occurrence. Therefore, we can speculate that serum CXCL-8 might be a better candidate as a tumor biochemical marker for the diagnosis of CRC than the routine clinical practice markers CEA and CA19-9.

Analysis of the relationship between serum CXCL-8 concentrations and clinicopathological features indicated that serum levels of CXCL-8 were significantly associated with distant metastasis and tumor size, which was further verified by Spearman's correlation test. Rubie et al. [31] also evaluated the association between serum CXCL-8 levels and the clinicopathological characteristics of CRC, and they found that CXCL-8 was highly overexpressed in CRC tissues, which correlated with the depth of tumor invasion and tumor size [32, 33]. These findings also indicate that serum CXCL-8 may be an additional prognostic biomarker for CRC.

However, this study had its limitations. First, this study had a single-center retrospective design that might have caused deviation toward subject selection and analysis. Moreover, we failed to obtain overall survival information for CRC; therefore, the correlation between serum CXCL-8 and overall survival for CRC was not assessed. Therefore, prospective multicenter studies with large sample sizes are required to assess the clinical utility of serum CXCL-8 levels for CRC diagnosis and its correlation with overall survival.

## 5. Conclusions

In summary, our results suggest that serum CXCL-8 may be a better biochemical tumor marker for CRC diagnosis than routine clinical blood-based markers or may be a suitable adjunct. Notably, we found a significant association between serum CXCL-8 levels and the clinicopathological features of patients with CRC, suggesting its use as a prognostic marker.

## Figures and Tables

**Table 1:** Statistical values of CXCL-8, CEA, and CA19-9 alone and combined for the differential diagnosis between patients with colorectal cancer and healthy participants.

Variable	AUC	Accuracy (%)	Cut-off value	Sensitivity (%)	Specificity (%)	95% Confidence interval	
						Lower limit	Upper limit
CXCL-8	0.920	84.57	24.92	86.34	81.30	89.24	94.80
CEA	0.837	57.43	5	34.80	99.19	75.59	87.88
CA19-9	0.730	45.43	37	15.86	100	67.81	78.26
CXCL-8 + CEA	0.954	85.14		78.41	97.56	93.51	97.22
CXCL-8 + CA19-9	0.940	84.86		82.82	88.62	91.62	96.26
CEA + CA19-9	0.844	77.43		74.45	82.93	80.34	88.41

**Table 2:** Statistical values of CXCL-8, CEA, and CA19-9 alone and together for the differential diagnosis between patients with colorectal cancer and patients with colorectal adenoma.

Variable	AUC	Accuracy (%)	Cut-off value	Sensitivity (%)	Specificity (%)	95% Confidence interval	
						Lower limit	Upper limit
CXCL-8	0.774	77.15	24.92	86.34	58.18	71.51	83.35
CEA	0.760	54.89	5	34.80	96.36	70.82	81.04
CA19-9	0.686	42.43	37	15.86	97.27	62.67	74.49
CXCL-8 + CEA	0.804	73.59		74.01	72.73	75.71	85.10
CXCL-8 + CA19-9	0.768	73.88		77.53	66.36	71.36	82.19
CEA + CA19-9	0.767	72.41		71.81	73.64	71.67	81.70

**Table 3:** Performance of CXCL-8, CEA, and CA19-9 for predicting the colorectal cancer occurrence risk.

Variables	No. subjects	Cut-off value	Multivariate analysis		
			OR	95% CI	*p* value
CEA (ng/mL)	298	<5	1 (R)	—	
	79	≥5	13.83	3.11 − 61.50	0.001

CA19-9 (U/mL)	340	<37	1 (R)	—	
	37	≥37	7.96	0.97 − 65.13	0.053

CXCL-8 (pg/mL)	135	<24.92	1 (R)	—	
	242	≥24.92	7.76	4.06 − 14.86	<0.001

**Table 4:** Relationship between the serum CXCL-8, CEA, and CA19-9 levels and the clinicopathological features of patients with colorectal cancer.

Clinicopathological characteristics	No. subjects	CXCL-8 (pg/mL)	CEA (ng/mL)	CA19-9 (U/mL)
*Location*				
Colon	122	37.58 (27.36–55.68)	3.90 (2.20–8.63)	14.28 (7.98–27.09)
Rectum	105	37.75 (29.12–62.14)	3.00 (1.80–6.80)	14.17 (8.48–23.74)
*p* value		0.458	0.111	0.580
*TNM stage*				
I	42	37.66 (28.56–56.82)	2.60 (1.48–4.75)	13.78 (7.62–23.59)
II	83	36.27 (27.38–62.06)	3.20 (2.00–8.60)	13.36 (7.50–25.86)
III	85	36.83 (28.12–56.98)	3.70 (2.10–7.75)	14.36 (9.40–24.45)
IV	17	53.38 (35.24–71.14)	8.40 (4.75–38.50)	22.71 (12.11–109.40)
*p* value		0.423	**<0.001**	0.073
*T stage*				
T1	11	38.98 (33.83–67.80)	1.50 (1.20–2.90)	11.21 (1.33–19.96)
T2	34	35.96 (28.93–55.05)	2.92 (1.80–5.78)	14.30 (8.77–23.59)
T3	154	36.33 (27.38–55.38)	3.60 (2.08–8.40)	13.86 (7.98–25.87)
T4	28	41.61 (31.35–69.72)	6.65 (2.35–20.68)	20.12 (11.60–48.44)
*p* value		0.416	**0.001**	0.077
*N stage*				
N0	123	36.27 (27.42–54.69)	2.90 (1.80–5.70)	13.65 (7.75–24.39)
N1	60	47.16 (31.39–78.31)	3.90 (2.45–9.05)	13.94 (9.27–24.70)
N2	44	35.95 (28.16–51.60)	4.90 (2.00–11.65)	17.12 (11.05–27.54)
*p* value		0.062	**0.016**	0.260
*M stage*				
M0	206	36.33 (27.72–55.38)	3.20 (1.90–6.20)	13.91 (8.14–23.46)
M1	21	53.38 (37.52–65.47)	8.40 (4.20–28.52)	23.52 (12.72–77.58)
*p* value		**0.039**	**<0.001**	**0.007**
*Histological grade*				
High	6	39.52 (32.71–66.49)	3.25 (2.20-4.30)	15.83 (8.62-22.43)
Moderate	206	37.66 (27.92–57.85)	3.40 (1.90-7.60)	13.91 (8.00-25.04)
Low	15	40.94 (35.28–55.24)	4.70 (1.95-14.65)	22.71 (14.34-36.18)
*p* value		0.718	0.621	0.095
*Vascular invasion*				
Present	41	38.15 (26.34–56.93)	4.20 (2.20–8.70)	16.60 (9.16–3.19)
Absent	175	38.16 (28.69–64.66)	3.35 (1.90–7.20)	13.84 (7.87–23.49)
Unknown	11	35.97 (32.71–37.63)	4.15 (2.60–6.10)	19.11 (13.96–25.04)
*p* value		0.638	0.523	0.082
*Nerve invasion*				
Present	66	39.42 (28.85–76.51)	4.20 (2.60–14.40)	15.00 (9.16–35.27)
Absent	155	36.98 (27.63–53.46)	3.00 (1.80–5.95)	13.86 (7.83–22.76)
Unknown	5	56.11 (37.52–81.40)	3.00 (2.90–5.30)	19.96 (8.40–22.71)
*p* value		0.184	**0.007**	0.165
*Tumor size (cm)*				
<5	129	36.11 (27.72–53.54)	2.90 (1.80–5.70)	14.50 (8.14–23.46)
≥5	98	42.80 (29.02–76.20)	4.15 (2.40–9.40)	13.84 (8.80–32.91)
*p* value		**0.034**	**0.009**	0.294

Data are presented as the median with the interquartile range and are statistically significant at *p* < 0.05 (bold).

**Table 5:** Spearman's rank correlations between the serum concentrations of CXCL-8, CEA, and CA19-9 and the clinicopathological characteristics among patients with colorectal cancer.

		TNM stage	T stage	N stage	M stage	Tumor size	Histological grade	Vascular invasion	Nerve invasion	CEA	CA19-9	CXCL-8
TNM stage	*r*	1.000	**0.678** ^∗∗^	**0.875** ^∗∗^	**0.456** ^∗∗^	0.085	**0.146** ^∗^	**-0.151** ^∗^	**-0.274** ^∗∗^	**0.265** ^∗∗^	**0.136** ^∗^	0.075
	*p*		**<0.001**	**<0.001**	**<0.001**	0.102	**0.014**	**0.012**	**<0.001**	**<0.001**	**0.021**	0.131

T stage	*r*	**0.678** ^∗∗^	1.000	**0.446** ^∗∗^	**0.324** ^∗∗^	**0.158** ^∗∗^	**0.207** ^∗∗^	**-0.114** ^∗^	**-0.319** ^∗∗^	**0.244** ^∗∗^	**0.139** ^∗^	0.048
	*p*	**<0.001**		**<0.001**	**<0.001**	**0.009**	**0.001**	**0.044**	**<0.001**	**<0.001**	**0.018**	0.237

N stage	*r*	**0.875** ^∗∗^	**0.446** ^∗∗^	1.000	**0.400** ^∗∗^	-0.044	**0.143** ^∗^	**-0.128** ^∗^	**-0.179** ^∗∗^	**0.186** ^∗∗^	0.104	0.037
	*p*	**<0.001**	**<0.001**		**<0.001**	0.257	**0.016**	**0.027**	**0.003**	**0.002**	0.060	0.290

M stage	*r*	**0.456** ^∗∗^	**0.324** ^∗∗^	**0.400** ^∗∗^	1.000	0.029	0.109	-0.084	-0.097	**0.277** ^∗∗^	0.181	**0.117** ^∗^
	*p*	**<0.001**	**<0.001**	**<0.001**		0.334	0.050	0.103	0.072	**<0.001**	0.003	**0.039**

Tumor size	*r*	0.085	**0.158** ^∗∗^	-0.044	0.029	1.000	0.093	-0.012	**-0.188** ^∗∗^	**0.173** ^∗∗^	0.070	**0.122** ^∗^
	*p*	0.102	**0.009**	0.257	0.334		0.080	0.431	**0.002**	**0.005**	0.148	**0.034**

Histological Grade	*r*	**0.146** ^∗^	**0.207** ^∗∗^	0.143	0.109	0.093	1.000	0.099	-0.055	0.064	**0.125** ^∗^	0.001
	*p*	**0.014**	**0.001**	0.016	0.050	0.080		0.069	0.204	0.168	**0.030**	0.493

Vascular invasion	*r*	**-0.151** ^∗^	**-0.114** ^∗^	**-0.128** ^∗^	-0.084	-0.012	0.099	1.000	**0.231** ^∗∗^	-0.046	-0.055	0.028
	*p*	**0.012**	**0.044**	**0.027**	0.103	0.431	0.069		**<0.001**	0.247	0.203	0.335

Nerve invasion	*r*	**-0.274** ^∗∗^	**-0.319** ^∗∗^	**-0.179** ^∗∗^	-0.097	**-0.188** ^∗∗^	-0.055	**0.231** ^∗∗^	1.000	**-0.202** ^∗∗^	**-0.118** ^∗^	-0.067
	*p*	**<0.001**	**<0.001**	**0.003**	0.072	**0.002**	0.204	**<0.001**		**0.001**	**0.039**	0.159

CEA	*r*	**0.265** ^∗∗^	**0.244** ^∗∗^	**0.186** ^∗∗^	**0.277** ^∗∗^	**0.173** ^∗∗^	0.064	-0.046	**-0.202** ^∗∗^	1.000	**0.423** ^∗∗^	0.015
	*p*	**<0.001**	**<0.001**	**0.002**	**<0.001**	**0.005**	0.168	0.247	**0.001**		**<0.001**	0.409

CA19-9	*r*	**0.136** ^∗^	**0.139** ^∗^	0.104	**0.181** ^∗^	0.070	**0.125** ^∗^	-0.055	**-0.118** ^∗^	**0.423** ^∗∗^	1.000	-0.001
	*p*	**0.021**	**0.018**	0.060	**0.003**	0.148	**0.030**	0.203	**0.039**	**<0.001**		0.491

CXCL-8	*r*	0.075	0.048	0.037	**0.117** ^∗^	**0.122** ^∗^	0.001	0.028	-0.067	0.015	-0.001	1.000
	*p*	0.131	0.237	0.290	**0.039**	**0.034**	0.493	0.335	0.159	0.409	0.491	

^∗^ *p* < 0.05, ^∗∗^*p* < 0.01 (bold).

**Table 6:** All acronym and full name in the article.

Acronym	Full name	Acronym	Full name
CRC	Colorectal cancer	OR	Odds ratio
CXCL-8	C-X-C motif chemokine 8	ROC	Receiver operating characteristic
CEA	Carcinoembryonic antigen	TME	Tumor microenvironment
CA19-9	Carbohydrate antigen-19-9	CON	Healthy controls
CA	Colorectal adenoma	AUC	Area under the curve

## Data Availability

The data used to support the findings of this study are included within the article.

## References

[1] Global Burden of Disease Cancer Collaboration (2019). Global, regional, and national cancer incidence, mortality, years of life lost, years lived with disability, and disability-adjusted life-years for 29 cancer groups, 1990 to 2017: a systematic analysis for the global burden of disease study. *JAMA Oncologia*.

[2] Feigin V., GBD 2017 Causes of Death Collaborators (2018). Global, regional, and national age-sex-specific mortality for 282 causes of death in 195 countries and territories, 1980-2017: a systematic analysis for the global burden of disease study 2017. *Lancet*.

[3] Bray F., Ferlay J., Soerjomataram I., Siegel R. L., Torre L. A., Jemal A. (2018). Global cancer statistics 2018: GLOBOCAN estimates of incidence and mortality worldwide for 36 cancers in 185 countries. *CA: a Cancer Journal for Clinicians*.

[4] Chen W., Zheng R., Baade P. D. (2016). Cancer statistics in China, 2015. *CA: a Cancer Journal for Clinicians*.

[5] Toiyama Y., Tanaka K., Inoue Y., Mohri Y., Kusunoki M. (2016). Circulating cell-free microRNAs as biomarkers for colorectal cancer. *Surgery Today*.

[6] Uraoka T., Hosoe N., Yahagi N. (2015). Colonoscopy: is it as effective as an advanced diagnostic tool for colorectal cancer screening?. *Expert Review of Gastroenterology & Hepatology*.

[7] Dekker E., Rex D. K. (2018). Advances in CRC prevention: screening and surveillance. *Gastroenterology*.

[8] Thomas D. S., Fourkala E. O., Apostolidou S. (2015). Evaluation of serum CEA, CYFRA21-1 and CA125 for the early detection of colorectal cancer using longitudinal preclinical samples. *British Journal of Cancer*.

[9] Grivennikov S. I. (2013). Inflammation and colorectal cancer: colitis-associated neoplasia. *Seminars in Immunopathology*.

[10] Hotchkiss R. S., Moldawer L. L. (2014). Parallels between cancer and infectious disease. *The New England Journal of Medicine*.

[11] Mantovani A., Allavena P., Sica A., Balkwill F. (2008). Cancer-related inflammation. *Nature*.

[12] Balkwill F. R. (2012). The chemokine system and cancer. *The Journal of Pathology*.

[13] Qazi B. S., Tang K., Qazi A. (2011). Recent advances in underlying pathologies provide insight into interleukin-8 expression-mediated inflammation and angiogenesis. *International Journal of Inflammation*.

[14] Xie K. (2001). Interleukin-8 and human cancer biology. *Cytokine & Growth Factor Reviews*.

[15] Lukaszewicz-Zajac M., Paczek S., Muszynski P., Kozlowski M., Mroczko B. (2019). Comparison between clinical significance of serum CXCL-8 and classical tumor markers in oesophageal cancer (OC) patients. *Clinical and Experimental Medicine*.

[16] Gong D., Li Z., Ding R. (2019). Extensive serum biomarker analysis in patients with nasopharyngeal carcinoma. *Cytokine*.

[17] Litman-Zawadzka A., Lukaszewicz-Zajac M., Gryko M., Kulczynska-Przybik A., Mroczko B. (2018). Serum chemokine CXCL8 as a better biomarker for diagnosis and prediction of pancreatic cancer than its specific receptor CXCR2, C-reactive protein, and classic tumor markers CA 19-9 and CEA. *Polish Archives of Internal Medicine*.

[18] Fridman W. H., Pages F., Sautes-Fridman C., Galon J. (2012). The immune contexture in human tumours: impact on clinical outcome. *Nature Reviews. Cancer*.

[19] Najdaghi S., Razi S., Rezaei N. (2020). An overview of the role of interleukin-8 in colorectal cancer. *Cytokine*.

[20] Edge S. B., Compton C. C. (2010). The American Joint Committee on Cancer: the 7th edition of the AJCC cancer staging manual and the future of TNM. *Annals of Surgical Oncology*.

[21] Sun Z. Q., Xu Y. Y. (2018). *Medical Statistics, [M.S.]*.

[22] Bewick V., Cheek L., Ball J. (2005). Statistics review14: logistic regression. *Critical Care*.

[23] Garborg K. (2015). Colorectal cancer screening. *The Surgical Clinics of North America*.

[24] Karin N. (2018). Chemokines and cancer: new immune checkpoints for cancer therapy. *Current Opinion in Immunology*.

[25] Asfaha S., Dubeykovskiy A. N., Tomita H. (2013). Mice that express human interleukin-8 have increased mobilization of immature myeloid cells, which exacerbates inflammation and accelerates colon carcinogenesis. *Gastroenterology*.

[26] Heiss J. A., Brenner H. (2017). Epigenome-wide discovery and evaluation of leukocyte DNA methylation markers for the detection of colorectal cancer in a screening setting. *Clinical Epigenetics*.

[27] Vatandoost N., Ghanbari J., Mojaver M. (2016). Early detection of colorectal cancer: from conventional methods to novel biomarkers. *Journal of Cancer Research and Clinical Oncology*.

[28] Song Y., Huang Z., Kang Y. (2018). Clinical usefulness and prognostic value of red cell distribution width in colorectal cancer. *BioMed Research International*.

[29] Zhang S. Y., Lin M., Zhang H. B. (2015). Diagnostic value of carcinoembryonic antigen and carcinoma antigen 19-9 for colorectal carcinoma. *International Journal of Clinical and Experimental Pathology*.

[30] Paczek S., Lukaszewicz-Zajac M., Gryko M., Mroczko P., Kulczynska-Przybik A., Mroczko B. (2020). CXCL-8 in preoperative colorectal cancer patients: significance for diagnosis and cancer progression. *International Journal of Molecular Sciences*.

[31] Rubie C., Frick V. O., Pfeil S. (2007). Correlation of IL-8 with induction, progression and metastatic potential of colorectal cancer. *World Journal of Gastroenterology*.

[32] Ueda T., Shimada E., Urakawa T. (1994). Serum levels of cytokines in patients with colorectal cancer: possible involvement of interleukin-6 and interleukin-8 in hematogenous metastasis. *Journal of Gastroenterology*.

[33] Baier P. K., Eggstein S., Wolff-Vorbeck G., Baumgartner U., Hopt U. T. (2005). Chemokines in human colorectal carcinoma. *Anticancer Research*.

